# The Use and Utility of Wearable Devices for Tracking Sleep and Activity in Inpatient Mental Health Settings: Protocol for a Rapid Review

**DOI:** 10.2196/82290

**Published:** 2026-03-10

**Authors:** Gillian Strudwick, Hwayeon Danielle Shin, Iman Kassam, Karishini Ramamoorthi, Benjamin Rosen, Michelle A Patriquin, Justin Baker, Nicholas Neufeld

**Affiliations:** 1 Campbell Family Mental Health Research Institute Centre for Addiction and Mental Health Toronto, ON Canada; 2 Institute of Health Policy, Management and Evaluation University of Toronto Toronto, ON Canada; 3 Sinai Health Toronto, ON Canada; 4 John S. Dunn Behavioral Sciences Center The University of Texas Health Science Center at Houston Houston, TX United States; 5 Institute for Technology in Psychiatry McLean Hospital Belmont, MA United States

**Keywords:** mental health, wearables, actigraphy, sleep, physical activity, rapid review

## Abstract

**Background:**

Wearable devices offer an opportunity to objectively monitor and capture sleep and activity in psychiatric inpatient settings, where traditional approaches often rely on subjective reporting or staff observation, both of which have inherent flaws. Technologies such as Fitbit, Garmin, Oura Ring, GENEActiv, Empatica, and WHOOP (among others) have been used to passively collect physiological data that may inform care planning and clinical decision-making. Despite growing interest, the extent to which these wearables are feasible, acceptable, and useful in inpatient mental health settings remains unclear. Synthesizing this evidence is essential to inform their potential integration into psychiatric care.

**Objective:**

This study aims to conduct a rapid review to examine the current use, utility, feasibility, and implementation of wearable devices for tracking sleep and/or activity in inpatient mental health settings. To date, no review has examined inpatient settings specifically.

**Methods:**

A rapid review will be conducted using Cochrane Rapid Review methods. Peer-reviewed literature will be searched in four databases: (1) PubMed, (2) Embase, (3) PsycInfo, and (4) CINAHL. Studies will be included if they report on the use of wearable devices in inpatient psychiatric settings and evaluate outcomes related to feasibility, acceptability, clinical utility, or implementation. Data extraction will be conducted using a standardized extraction template, and findings will be synthesized narratively and via descriptive statistics.

**Results:**

Database searches are currently underway. The review is expected to be completed by June 2026. A manuscript detailing the findings of this review will be published thereafter.

**Conclusions:**

This rapid review will provide timely evidence to support the integration of wearable technologies into psychiatric inpatient settings. Findings will inform future research and clinical practice focused on the use of wearables for sleep and activity monitoring, contributing to more responsive and data-informed mental health care.

**International Registered Report Identifier (IRRID):**

PRR1-10.2196/82290

## Introduction

Accurate monitoring of sleep and physical activity in psychiatric inpatient settings is essential for understanding patients’ mental health status and treatment response [1]. Sleep disturbances such as insomnia, hypersomnia, and fragmented sleep are highly prevalent across psychiatric conditions such as depression, bipolar disorder, schizophrenia, and anxiety, serving both as core features of these disorders and potential triggers for relapse [2]. Poor sleep quality in psychiatric inpatients has been linked to worse clinical outcomes, including greater emotion regulation difficulties, elevated depression and anxiety, increased suicidal ideation, and more severe nightmares [3-5]. Similarly, low levels of physical activity are associated with poorer mental health, whereas increased activity can enhance emotional regulation and cognitive function and reduce depressive and anxiety symptoms [6]. As both sleep and activity may signal shifts in mental state, objective and continuous assessment of these domains is important in inpatient settings where patients may lack self-awareness or the capacity to reliably self-report, enabling timely, data-driven clinical interventions.

Traditional methods of assessing or monitoring sleep and activity, such as self-report or staff observation (often by nurses), are often subjective and imprecise [7-9]. These approaches can be limited by recall bias, inconsistency across observers, and challenges in obtaining continuous or real-time data [10]. In settings where clinical acuity is high and patients may be experiencing cognitive or emotional distress, reliable and objective data are required for care such as tailoring care plans, identifying early signs of deterioration, and evaluating treatment effectiveness over time [11].

Wearable technologies offer a promising solution [12] by providing passive, continuous, and generally noninvasive monitoring of sleep and physical activity (among other physiological measures) via a wrist-worn device or ring [13,14]. Devices, including but not limited to Fitbit (Google Inc), Garmin (Garmin Ltd), Oura Ring (Oura Health Ltd), GENEActiv (Activinsights Ltd), Empatica (Empatica Inc), and WHOOP (Whoop, Inc) can generate physiological data that may enhance clinical decision-making and have the potential to reduce clinician documentation burden [15]. In addition to supporting personalized interventions and early detection of changes in mental health status, wearables have the potential to empower patients by increasing engagement with and awareness of their health patterns (eg, sleep length, sleep quality, and step count) [14,16]. However, the utility and implementation of these devices in the context of inpatient psychiatry, where safety concerns, patient acuity, organizational policies, and technology infrastructure differ from outpatient or nonpsychiatric settings, are not well established. Understanding how these technologies are currently being used and whether they are feasible, acceptable, and clinically useful in inpatient environments is an essential step toward integrating them effectively into mental health care [17].

Given the increasing interest in digital health tools in psychiatry and the limited evidence specific to inpatient settings, there is a need to understand how wearables are currently being used and evaluated in these environments. Inpatient psychiatric settings present unique challenges such as restricted environments, high patient acuity, unique privacy concerns, increased vulnerability of patients, and at times limited digital infrastructure [18,19]. Despite these challenges, wearables could offer important benefits: passive data collection, reduced burden on clinical staff, and early detection of symptom exacerbation or treatment effect [12,20]. Synthesizing the current literature on this topic is necessary to identify best practices, feasibility, and gaps in the literature that can guide future implementation and research. A rapid review will therefore be conducted with the following research questions (RQs):

- RQ1: How are wearable devices being used to monitor sleep and/or activity in inpatient psychiatric settings?
- RQ2: What is the reported feasibility, acceptability, and clinical utility of using wearable technologies in inpatient mental health care?

This review will differ from previous reviews of wearable technologies in mental health, which have synthesized studies primarily in outpatient or community settings and have focused on identifying associations between passively collected data and psychiatric symptoms or relapse risk. Previous reviews have also been predominantly conducted for research purposes, while this review will include both research purposes and real-world use. This protocol focuses specifically on inpatient mental health settings, where patient acuity, safety requirements, and clinical workflows introduce distinct considerations for technology use.

## Methods

This rapid review will be conducted following the methodological guidance provided by the Cochrane Rapid Reviews Group [21] and the approach described by Tricco et al [22,23]. A rapid review design was selected due to its ability to generate timely insights in a field where the evidence base is still emerging [24]. This rapid review will not include a critical appraisal within its scope, as this is not common practice [25,26] and would extend timelines. The process as described balances efficiency with methodological rigor and will take place over a 6-month timeline.

The objective of this review is to explore the use, utility, feasibility, and implementation of wearable devices for tracking sleep and/or activity in inpatient psychiatric settings. The review will address 3 primary RQs described earlier.

Studies will be eligible for inclusion if they are primary empirical research (quantitative, qualitative, or mixed methods); if they are conducted in inpatient psychiatric or mental health settings; and if they include the use of wearable technologies such as Fitbit, Garmin, Oura Ring, GENEActiv, Empatica, or WHOOP (or other similar devices) to monitor sleep and/or activity. For this review, inpatient psychiatric or mental health settings will include any facility providing mental health care support or treatment to patients staying within that facility for the duration of their treatment. Included studies must report outcomes related to feasibility, acceptability, clinical utility, or implementation. Feasibility refers to the practicality and achievability of using wearables in a clinical environment, including factors such as cost, workload for staff, and technical and infrastructural requirements. Acceptability assesses the attitudes and willingness of both patients and clinicians to use the technology. The concept of clinical utility focuses on whether the data collected from wearables provide meaningful and actionable insights that can inform patient care (eg, care planning), detect changes, and evaluate treatment effectiveness. Studies will be excluded if they focus solely on outpatient settings or nonpsychiatric mental health inpatient areas. Only published peer-reviewed articles will be considered, and no restrictions will be placed on publication year, although it is likely that articles identified will be primarily in the last 5 to 10 years given the popularity and availability of these wearables during this period.

A comprehensive search strategy was developed (refer to Multimedia Appendix 1 showing the search strategy for PubMed, which will be translated to the other databases) and was peer-reviewed using the Peer Review of Electronic Search Strategies (PRESS) guideline [27]. The literature will be identified from four databases: (1) PubMed, (2) Embase, (3) PsycInfo, and (4) CINAHL. The search uses a combination of subject headings and keywords relating to wearable devices, sleep and activity monitoring, and inpatient psychiatric care.

All references identified from the databases will be imported into Covidence (Veritas Health Innovation Ltd) to manage duplicates and facilitate screening. Titles and abstracts will be screened by one reviewer, with the first 100 citations checked by a second reviewer for consistency. If 90% or more agreement is reached by the 2 reviewers, full-text screening will proceed. If not, the reviewers will meet to discuss discrepancies and carry on to another 100 citations until 90% agreement is reached. There is no predefined limit, and the screening will continue until 90% agreement is reached. Cohen κ will be reported as the measure of agreement. Full-text screening will be conducted independently by 2 reviewers, with disagreements resolved through discussion or consultation with a member of the review team as needed. The selection process will be documented using a PRISMA (Preferred Reporting Items for Systematic Reviews and Meta-Analyses) flow diagram [28].

Data extraction will be carried out using a data extraction form, which will be pilot tested initially. One reviewer will extract the data, and a second reviewer will verify their accuracy. Extracted data will include basic study details (authors, title, etc); study characteristics (sample size, etc); type and version of wearable device; purpose of use; duration; outcomes monitored and assessed; and findings related to feasibility, acceptability, and utility. Additionally, ethical, legal, and safety aspects will be systematically extracted and synthesized as outcome domains. For each RQ, data will be characterized to address each review question. Both quantitative and qualitative findings will be brought together in a narrative structure. Descriptive statistics will be used to report discrete data, and all identified data will be accompanied by appropriate visualizations, narrative descriptions, and tables as necessary. For the thematic analysis, thematic findings will be identified using inductive analysis of the data. For the extraction form, pilot testing of the tool will be carried out using pilot studies to ensure consistency of the data extraction process between reviewers, and necessary modifications will be made to ensure alignment among the reviewers.

To maximize resources, our protocol will include 2 reviewers working to code data in tandem. As needed, a senior team member will be consulted to resolve any disagreement between the coders.

To validate findings and enhance interpretation, the research team will engage in a consultation process with key knowledge users. These consultations will include clinicians, researchers, individuals with lived experience of inpatient psychiatric care, and digital health leaders. Consultations will be conducted virtually and will aim to validate the results, identify implications for clinical practice and policy, and help guide future research directions. Feedback obtained will be integrated into the Discussion section of the final manuscript.

No ethics approval is required for this rapid review, as it involves the analysis of secondary data from published sources.

## Results

The review will be conducted for a 6-month period and is anticipated to conclude in June 2026. In the first 2 months, the review team will translate the PubMed search strategy to other databases, carry out the database searches, and screen titles and abstracts using the eligibility criteria. In the third and fourth months, full-text articles will undergo review, and if they meet the inclusion criteria, data extraction will occur. In the fifth and sixth months, a narrative synthesis of the findings will be conducted. We will report the results of the searches and selection process via a PRISMA flow diagram. Data will be presented both quantitatively (device types used, common outcomes measured, etc) and qualitatively (themes related to feasibility, acceptability, implementation factors, etc). Results will be structured in alignment with the 3 RQs and key gaps in the literature will be highlighted. Knowledge user consultations will also occur in the final month, during which early synthesized findings will be shared and feedback will be obtained. We anticipate that these consultations will consist of in-person and virtual meetings with clinicians, researchers, and patient advisers who have or who plan to use wearables in their research or clinical care. We will plan to connect with the authors of up to 5 of the studies included in the review, as well as 5 people with lived experience and 5 clinicians from both medicine and nursing. The insights will enhance the contextual relevance of the review and guide the framing of the discussion for the final paper describing the findings. These numbers were selected to both manage feasibility and provide a breadth of opinions and insights. While formal research ethics board approval is not required for this review, we will obtain approval from our quality projects ethics review committee for the consultations that we plan to conduct.

## Discussion

### Anticipated Findings

This rapid review is expected to synthesize and clarify how wearable devices are currently being used to monitor sleep and physical activity in inpatient psychiatric settings and to summarize the reported feasibility, acceptability, and clinical utility of these technologies in real-world inpatient contexts. On the basis of preliminary scoping and prior literature in adjacent settings, we anticipate that the findings will show that wearable devices are primarily used for research and observational purposes, with more limited examples of routine clinical integration into inpatient care processes. We further expect that feasibility and acceptability will be reported as generally favorable among many patient populations, particularly when devices are noninvasive, easy to use, and supported by staff; however, we also anticipate that important challenges related to safety policies, data governance, staffing capacity, and integration into clinical workflows may emerge. Regarding clinical utility, it is anticipated that most studies will report potential or perceived value rather than direct evidence of changes in clinical decision-making or patient outcomes. The review may therefore highlight a gap between the promise of wearable-derived data and its actionable use in inpatient psychiatric care.

Previous reviews of wearable technologies in mental health have largely focused on outpatient, community, or ambulatory populations, with an emphasis on associations between passively collected physiological data and psychiatric symptoms, relapse risk, or functional outcomes. These reviews have demonstrated growing interest in consumer-grade wearables and their potential to support mental health research and care, but they have provided limited insight into the unique realities of inpatient psychiatric environments. By focusing on inpatient mental health settings, this review addresses a gap in the literature. Inpatient environments differ from outpatient settings in patient acuity, safety requirements, environmental constraints, and clinical workflows. As a result, findings from outpatient studies may not be directly transferable but still may provide some important and interesting insights.

Findings from this rapid review will be disseminated through publication in a peer-reviewed journal and presentations at relevant national and international conferences. In addition, results will be shared with clinicians, health system leaders, and individuals with lived experience through targeted knowledge translation activities, including presentations, summaries, and stakeholder meetings. Where feasible, accessible summaries will be developed to support broader uptake and inform decision-making related to the use of wearable technologies in inpatient psychiatric care.

### Conclusions

This rapid review will generate timely, practical insights into the current use and utility of wearable devices for tracking sleep and activity in inpatient psychiatric settings. By synthesizing evidence across different available wearable technologies and clinical contexts, the review will identify the feasibility, acceptability, and clinical utility of these tools, as well as common barriers and facilitators to implementation. Findings will provide a foundation for future clinical integration and research, particularly in tailoring digital health innovations to meet the needs of complex inpatient populations [29]. Ultimately, this work aims to support the development of safer, more responsive, and data-informed mental health care environments.

## Appendix

Multimedia Appendix 1 PubMed search strategy conducted on August 7, 2025.

## Abbreviations

PRESS: Peer Review of Electronic Search Strategies
PRISMA: Preferred Reporting Items for Systematic Reviews and Meta-Analyses
RQ: research question
