# Supplementary material for: The Use and Utility of Wearable Devices for Tracking Sleep and Activity in Inpatient Mental Health Settings: Protocol for a Rapid Review
Source: JMIR Res Protoc. 2026 Mar 10;15:e82290. doi: 10.2196/82290 (PMC13014072; doi:10.2196/82290)
Supplement: Multimedia Appendix 1 [file resprot_v15i1e82290_app1.docx]

**Multimedia Appendix 1:** Draft Search Strategy for PubMed Created On Aug 7^th^, 2025

| **Concept** | **Query #** | **Search Terms** | **Results** |
| --- | --- | --- | --- |
| Wearables  This includes activity and sleep | 1 | "Accelerometry"[Mesh] OR “acceleromet*”[tw] | 32,679 |
|  | 2 | "Fitness Trackers"[Mesh] OR "fitness tracker"[tw] OR "fitness trackers"[tw] OR "fitness tracking"[tw] | 1,850 |
|  | 3 | "activity monitor*"[tw] OR "activity tracker*"[tw] OR "activity tracking"[tw] | 7,032 |
|  | 4 | “Wearable electronic devices”[MESH] OR “wearable electronic device*”[tw] OR “wearable device*”[tw] OR “wearable technolog*”[tw] OR “wearable sensor*”[tw] OR “wearable monitor*”[tw] | 36,961 |
|  | 5 | “Smartphone”[MESH] OR “smartwatch*”[tw] OR “smart ring*”[tw] OR “oura ring”[tw] OR “fitbit”[tw] OR "garmin"[tw] OR "misfit shine"[tw] OR “whoop”[tw] OR "polar loop"[tw] OR "jawbone*"[tw] OR “Samsung”[tw] OR “geneactiv”[tw] OR “empatica”[tw] OR “mio”[tw] OR “actigraph*”[tw] OR "apple watch*"[tw] OR “withings”[tw] OR “pebble”[tw] OR “sensewear”[tw] OR “pedometer*”[tw] | 36,817 |
|  | 6 | OR 1-5 | 95,974 |
| Setting; in-patient | 7 | “hospitalis*”[tiab] OR “hospitaliz*”[tiab] OR “inpatient*”[tiab] OR “in-patient*”[tiab] | 2,770,509 |
|  | 8 | “admission*”[tiab] OR “admit*”[tiab] OR “hospital*”[tiab] OR “institution*”[tiab] OR “unit*”[tiab] OR “ward*”[tiab] | 3,459,630 |
|  | 9 | “mental*”[tiab] OR “psychiatr*”[tiab] AND (facilit*[tiab] OR institution*[tiab] OR unit*[tiab] OR ward*[tiab]) | 110,154 |
|  | 10 | "Hospitalization"[Mesh] OR "Inpatients"[Mesh] | 343,330 |
|  | 11 | OR 7-10 | 5,457,272 |
| Psychiatry/mental health | 12 | "Mental Health"[Mesh] OR "Mental Disorders"[Mesh] OR "Psychiatry"[Mesh] OR “mental health”[tiab] OR “mental illness*”[tiab] OR “mental disorder*”[tiab] OR “psychiatric”[tiab] OR “psychiatry”[tiab] OR “psychological”[tiab] OR “psychosocial”[tiab] OR “behavioural health”[tiab] OR “behavioral health”[tiab] | 2,239,521 |
|  | **13** | **6 AND 11 AND 12** | **2,560 results** |
